# 1842. Low Birthweight and Retention in HIV Care among Postpartum Women Living with HIV in Ghana

**DOI:** 10.1093/ofid/ofad500.1670

**Published:** 2023-11-27

**Authors:** Noora Kazanji, Kwame Sakyi, Bianca Edler

**Affiliations:** Corewell Health, Bloomfield Hills, Michigan; Oakland University, Rochester, Michigan; Oakland University, Rochester, Michigan

## Abstract

**Background:**

In Ghana, caring for small babies is associated with low social support, delayed care seeking, and experience of perceived stigma. These psychosocial factors can affect retention in HIV care for mothers living with HIV (MHIV). This has implications for maternal health and continuous access to lifesaving antiretroviral therapy (ART). Consequently, these women have higher risks of mortality, disease progression, and elevated viral load which can place the infant at risk of HIV acquisition through breastfeeding. The goal of this study was to examine the effect of infant birthweight on retention in HIV care among postpartum MHIV in Ghana.

**Methods:**

A secondary analysis was conducted on a longitudinal study which examined how caring for low birthweight infants influenced maternal ART adherence. Study participants included 126 postpartum MHIV who delivered at hospitals in Accra, Ghana. Participants were enrolled at time of birth and followed for six months. Modified Poisson and Poisson Bivariate and Multivariate regression models were performed to examine two primary outcomes: missing one or more of the three expected visits for HIV care within six months after birth and total number of HIV clinic days missed within six months after birth. Primary exposure variables included hospital recorded birthweight and the size of the infant as perceived by the mother.

**Results:**

In multivariate analysis, controlling for ART adherence, time on ART, education, income, baby health, age, hospital site, and internalized HIV stigma, for every 1 unit increase in birthweight, MHIV missed significantly less days of HIV care [IRR = 0.71, CI: 0.66 - 0.8, p< 0.001]. MHIV who perceived their infant size to be less than average missed significantly more visits when compared to MHIV who perceived their infant size to be average, while controlling for the same covariates [IRR = 1.41, CI: 1.27- 1.56, p< 0.001].

**Figure:**
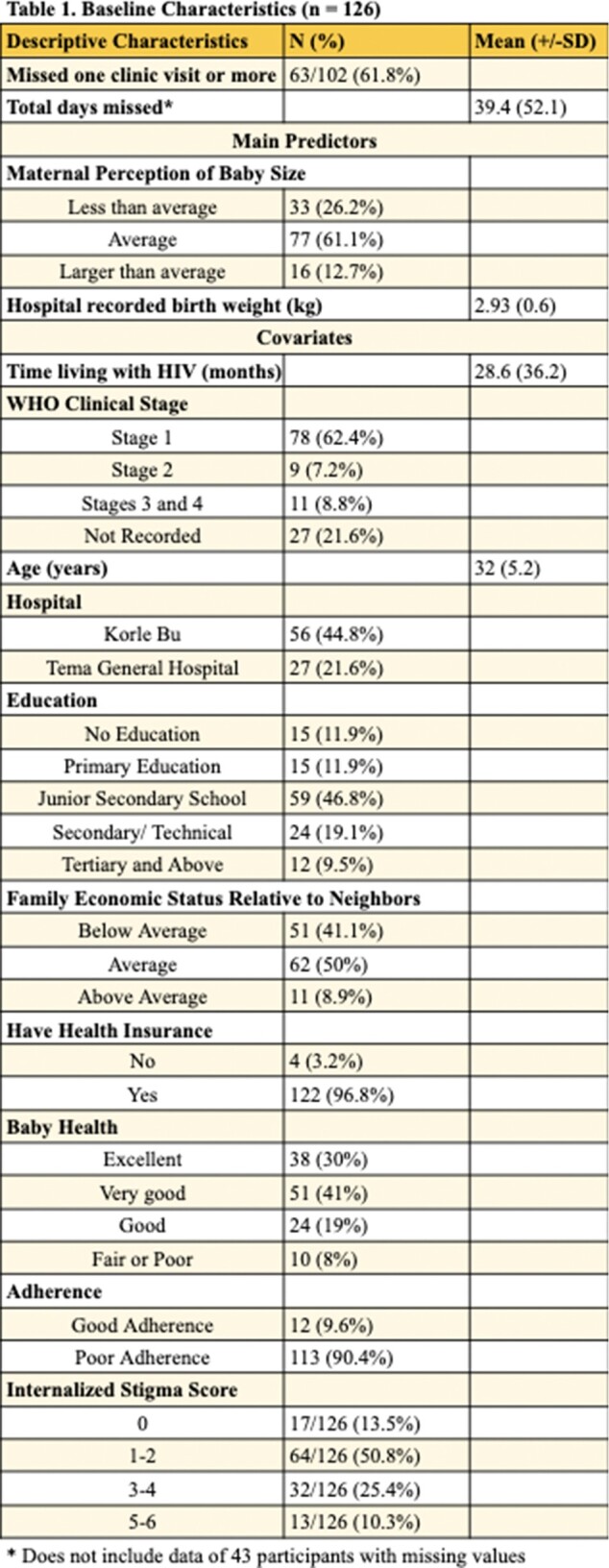

**Figure:**
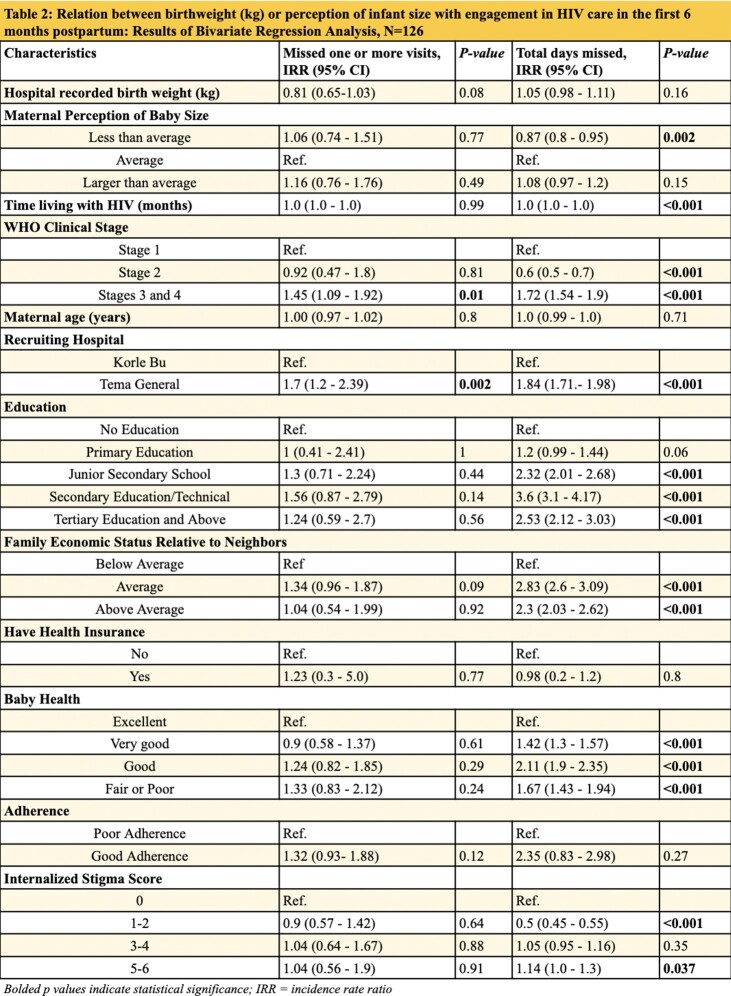

**Figure:**
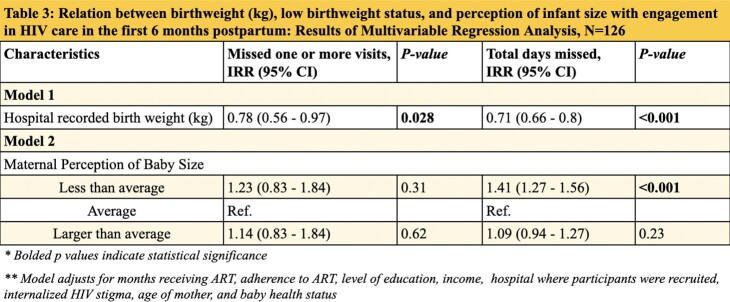

**Conclusion:**

For MHIV, the size of the baby affects retention in HIV care from both hospital-recorded birthweight and perceived infant size by the mother. Further interventions are needed at the individual and health system levels, especially towards reducing internalized stigma, to help mothers remain engaged in HIV care.

**Disclosures:**

**All Authors**: No reported disclosures

